# The Influence of a Novel, Crenelated Design of CAD-CAM Ceramic Veneers on the Debonding Strength

**DOI:** 10.3390/ma16103694

**Published:** 2023-05-12

**Authors:** Alexandra-Cristina Măroiu, Anca Jivănescu, Dan-Andrei Șerban, Radu-Marcel Negru, Virgil-Florin Duma, Cosmin Sinescu, Mihai Romînu

**Affiliations:** 1 Research Center in Dental Medicine Using Conventional and Alternative Technologies, School of Dental Medicine, “Victor Babes” University of Medicine and Pharmacy of Timisoara, 9 Revolutiei 1989 Ave., 300070 Timisoara, Romania; maroiualexandra@gmail.com (A.-C.M.); minosinescu@gmail.com (C.S.); mrominu@hotmail.com (M.R.); 2 Department of Prosthodontics, “Victor Babes” University of Medicine and Pharmacy, 9 Revolutiei 1989 Ave., 300070 Timisoara, Romania; 3 TADERP Research Center, 300041 Timisoara, Romania; 4 Faculty of Mechanics, Polytechnic University of Timisoara, 1 Mihai Viteazu Ave., 300222 Timisoara, Romania; dan.serban@upt.ro (D.-A.Ș.);; 5 3OM Optomechatronics Group, Faculty of Engineering, “Aurel Vlaicu” University of Arad, Str. Elena Dragoi No. 2, 310177 Arad, Romania; 6 Doctoral School, Polytechnic University of Timisoara, 1 Mihai Viteazu Ave., 300222 Timisoara, Romania; 7 Department of Prostheses Technology and Dental Materials, “Victor Babes” University of Medicine and Pharmacy, 9 Revolutiei 1989 Ave., 300070 Timisoara, Romania

**Keywords:** ceramic veneers, computer-aided design, computer-aided manufacturing, lithium disilicate, crenelated tooth preparation, sinusoidal design, adhesive forces, mechanical resistance, finite element analysis, aesthetic dentistry

## Abstract

(1) Background: Aesthetic dentistry has become one of the most dynamic fields in modern dental medicine. Ceramic veneers represent the most appropriate prosthetic restorations for smile enhancement, due to their minimal invasiveness and highly natural appearance. For long-term clinical success, accurate design of both tooth preparation and ceramic veneers is of paramount importance. The aims of this in vitro study were to assess the stress in anterior teeth restored with Computer-Aided Design (CAD) and Computer-Aided Manufacturing (CAM) ceramic veneers and compare the resistance to detachment and the fracture of ceramic veneers prepared using two different designs. (2) Methods: Sixteen lithium disilicate ceramic veneers were designed and milled using the CAD-CAM technology and divided into two groups according to the preparations (*n* = 8): Group 1, conventional (CO), with linear marginal contour and Group 2, crenelated (CR), the latter with our novel (patented) sinusoidal marginal design. All samples were bonded to anterior natural teeth. The mechanical resistance to detachment and fracture was investigated by applying bending forces on the incisal margin of the veneers in order to determine which type of preparation leads to better adhesion. An analytic method was employed, as well, and the results of the two approaches were compared. (3) Results: The mean values of the maximum force recorded at the veneer detachment were 78.82 ± 16.55 N for the CO group and 90.20 ± 29.81 N for the CR group. The relative increase, equal to 14.43%, demonstrated that the novel CR tooth preparation provided higher adhesive joints. In order to determine the stress distribution within the adhesive layer, a finite element analysis (FEA) was performed. The statistical *t*-test showed that the mean value of the maximum normal stresses is higher for the CR-type preparations. (4) Conclusions: The patented CR veneers represent a practical solution to augment the adhesion and mechanical properties of ceramic veneers. The obtained results demonstrated that CR adhesive joints triggered higher mechanical and adhesive forces, which subsequently led to a higher resistance to detachment and fracture.

## 1. Introduction

The overall aesthetics of a person’s smile greatly depend on the teeth in the aesthetic zone. Major caries lesions, large unsuccessful previous restorations, and trauma are the most frequent causes of anterior teeth abnormalities. Therefore, various direct and indirect restorative strategies must be employed in order to correct such issues. In this respect, for many patients, direct filling-based restorations provide a rapid and affordable treatment option. However, in addition to the high risk of recurrent caries, direct restorations have several drawbacks, such as long-term discoloration due to the composite aging process. Another popular treatment method for the previously mentioned diagnoses is based on full-crown restorations that encompass the entire tooth structure. As they require complete tooth coverage and may provide superior retention and aesthetics compared to direct fillings, crown restorations have historically been the first treatment option for numerous cosmetic issues. However, the process of teeth preparation for these restorations can be considered invasive because it frequently requires a major removal of a part of the healthy tooth structure. In order to overcome the aforementioned disadvantages, ceramic veneers were introduced in the early 1980s with the aim of providing both conservativeness and a highly natural appearance [1].

The long-term clinical success of ceramic veneers has been closely linked to several factors, such as tooth surface and morphology, ceramic thickness, type of luting agent and adhesive system, marginal and internal fit of the veneers to tooth surface, functional and parafunctional activities, as well as geometry of the preparation [2]. Numerous experimental studies have been conducted with the aim of improving both the mechanical and cosmetic properties of such veneers. The directions of research in this respect have been focused on topics such as materials and technological processes to fabricate veneers, luting agents and protocols of the veneers to the dental surface, as well as distinct tooth preparation for prospective ceramic veneers [3,4,5,6]. The choice between indirect composites and ceramic for veneer fabrication has been controversial as far as mechanical properties and cost-effectiveness issues are concerned. However, a systematic review recently indicated that a higher risk of failure was observed for indirect composites in comparison to ceramic veneer restorations [7].

Traditional feldspathic porcelain systems have been preferred due to their optical properties and the conservativeness of the dental structure. However, they display low mechanical properties; hence, the development of novel all-ceramic materials with increased fracture toughness was encouraged. A widely-utilized glass-ceramic system (both milled or pressed) is lithium disilicate, which can be utilized as a monolithic material for manufacturing various parts, from porcelain veneers to fixed dental prostheses in the posterior region. This area of application is based on the material’s good mechanical properties, biocompatibility, high flexural strength, and chemical stability [8,9,10,11].

Regarding tooth preparation, ceramic veneers are usually cemented by utilizing light-activated resin cements because they provide longer working time, color stability, as well as proper physical and biomechanical properties [12]. Alternatively, flowable composites can be utilized for the luting procedure because they have good color stability [12], with similar polymerization shrinkage and film thickness as compared to resin cements [13]. However, the absence of try-in pastes is a disadvantage [14,15].

Referring to the tooth preparation design for veneers, there are four types described in the literature: window, feather, bevel, and incisal overlap [5]. While there are minor differences between them, all these designs have in common the linear proximal contour that outlines the extent of the prospective veneer. The issue is that many cases of clinical failure due to veneer detachment from the dental support have been reported, and most have been caused by an inappropriate tooth preparation technique. This is caused by the fact that the conventional (CO) design, characterized by the linear marginal contour, has the drawbacks of a small contact surface with the adjacent enamel and a large amount of sound tooth removal [16,17]. This justified our approach of introducing a novel design, with a sinusoidal proximal design of the preparation, in order to enhance the interfacial adhesive forces and mechanical resistance to veneer detachment from subjacent enamel. This new type of dental veneer, which we have called “crenelated” (CR), is the subject of a patent of our group [18].

Another important aspect that must be considered is that extending the preparation into the dentin severely affects the long-term clinical success [19]. Therefore, all samples were bonded to enamel in our works. In order to diminish the failure rate due to veneer detachment, a lot of efforts have been made to develop adhesives and luting systems with higher physical and mechanical properties [4]. However, one should not rely on the adhesive mechanisms exclusively, but focus on exploring other possible conservative designs for tooth preparation, and we approached this in our previous studies [18,20,21].

Taking the aforementioned aspects into consideration, the main purpose of our direction of research was to develop such novel CR veneers. We introduced this concept in a preliminary experimental study that comparatively evaluated the adhesive forces between CO and CR veneers and resin models, by applying bending forces on the incisal margins [20]. Moreover, we assessed the marginal and internal fit of CR versus CO veneers to enamel tooth preparations by using optical microscopy and micro-computed tomography in a follow-up work [21]. The results of both studies revealed several advantages of CR veneers, namely: increased adhesive forces by more than 60%, which decrease the probability of restoration detachment; higher retention forces, due to the peripheral micro-retentions that form an intricate joint between the veneer and the substrate [20]; a higher marginal adaptation (60 μm) of the CR compared to the CO veneers (230 μm); considerably better internal adaptation for CR veneers in comparison to CO, as the clinically accepted cement thickness/width of up to 120 μm covered 81.5% of the tooth surface for CR compared to 64.5% for CO; better contact between the surface of the veneer and the tooth, thus combining both adhesive and mechanical forces in order to prevent the veneer detachment; a more accurate positioning of the veneers in situ during the luting procedure [21].

The aim of the present study is to assess the influence that such a CR design has on the interfacial adhesive forces and on the mechanical resistance to detachment of veneers in comparison to the CO design. As the veneer detachment from the dental structure occurs frequently in daily practice, mainly because of inappropriate tooth preparation and geometry, the novel patented CR design that we have proposed may provide better interfacial adhesive forces and higher mechanical resistance to detachment than the CO design. The sinusoidal marginal contour of the CR preparation creates micro-retentions into the enamel; therefore, it should be capable of augmenting both the contact surface for adhesion and the mechanical retention to the subjacent tooth.

## 2. Materials and Methods

The present study was conducted according to the guidelines of the Declaration of Helsinki. It was approved by the CECS no. 5/19.01.2021 of the Ethical Committee of the “Victor Babes” University of Medicine and Pharmacy of Timisoara, Romania.

### 2.1. Tooth Surface Preparation

**Figure 1:**
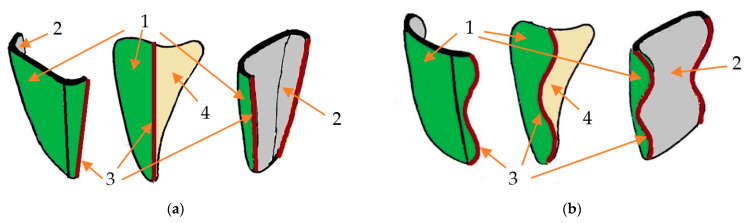
The two types of veneer design included in the experimental study: (**a**) conventional (CO), with linear proximal margins; (**b**) crenelated (CR), with sinusoidal proximal margins. Notations: 1, external surface of the veneer; 2, internal surface of the veneer; 3, proximal margins; 4, internal surface of the veneer, placed towards the tooth surface.

Sample size was determined based on Altman’s nomogram, as pointed out in the literature [2]. For a power of 80% and a standardized difference of approximately 1.18, a sample size *N* equal to 22 was obtained; therefore, *N*/2 for each group. During the testing, three specimens from each group failed in an inappropriate manner (i.e., by teeth fracturing), and were consequently excluded from study. Hence, sixteen human maxillary teeth (canines, central incisors, and lateral incisors), extracted for periodontal reasons, displaying no restorations or decays were finally utilized in the investigations. Calculus, dental plaque, and periodontal fibers were removed. Depending on the type of tooth preparation, the samples were divided into two experimental groups, each one consisting of three upper right central incisors, three upper right lateral incisors, and two upper right canines: Group 1, with CO veneers (Figure 1a) and Group 2, with CR veneers (Figure 1b), similar to [20]. In order to minimize the influence of the shape and size of the considered teeth on the results, the mesial-distal and facial-palatal dimensions of each sample were measured with a Digimatic Caliper 4 with a 0.1 mm accuracy (BEI Technologies Inc., Duncan Electronics Division, Irvine, CA, USA). Thus, only similar teeth, with less than 0.1 mm linear differences, were selected for and utilized in the study.

During all the stages of this work, the samples were stored in a 0.9% sodium-chloride physiologically sterile solution at 25 °C, and dehydration was avoided. To standardize the teeth preparation process, they were performed by the same author, using the same type of burs and the same 4× magnification system (Univet, Rezzato, Italy).

The CO dental preparation specific for Group 1 consisted of reducing the incisal margin by 1 mm and the buccal surface by 0.5 to 0.8 mm, tracing a linear proximal contour, with its limits positioned buccally to the interdental contact (Figure 1a).

The novel CR preparation specific for Group 2 displays sinusoidal proximal lines that outline the contour of the facial dental veneer (Figure 1b). The proximal limits were positioned buccally to the interdental contact. The height of the crenelated lines was correlated with the type of tooth, namely: 2 to 2.5 mm for lateral and inferior central incisors and 2.5 to 3 mm for central upper incisors and canines. The depth of the sinusoidal proximal margins decreased progressively from 0.6 to 0.8 mm in the gingival third, down to 0.4–0.6 mm in the middle third, and further down to 0.3–0.4 mm in the incisal third [18]. The buccal face of the tooth was reduced by 0.5 to 0.8 mm, and the incisal margin by 1 mm.

Thus, the CR dental veneers had a particular design that fits with the preparation of the tooth (Figure 1b): three sinusoidal proximal margins with different heights and depths, 0.5 to 0.8 mm facial thickness, and 1 mm incisal thickness. Even though the “dog-leg preparation” [5] describes the lingual extension of the proximal margin between the contact area and the gingival papilla (therefore creating a slightly convex aspect of the proximal tooth reduction), the novel patented crenelated preparation is characterized by two or three proximal sinusoidal lines (with specific characteristics depending on the tooth type and clinical case). This provides a more fluid substrate for the further luting procedure. The aim of these sinusoidal margins is to enhance the adhesion surface between enamel and ceramic, thus triggering higher debonding strength.

Both groups were characterized by a butt joint finish line situated at the incisal margin and a chamfer finish line along the cervical and proximal contour. The kit of diamond burs (Komet Dental, Lemgo, Germany) utilized for the tooth preparation design had coarse-grained cylindrical burs (0.8 mm and 1 mm in diameter), fine-grained cylindrical burs (0.8 mm and 1 mm in diameter), rounded-tip Arkansas stone burs, and super-fine polishing discs. The sequences of the tooth reduction and finishing protocols were the same for both types of veneers. First, the reduction of the incisal margin was performed. Second, the preparation of the buccal face was completed, while outlining the marginal contour. The proximal finishing lines were commonly extended just buccally to the interdental contact, except for clinical situations when the form and color of the tooth had to be changed or when there was a diastema that required closure. In these clinical contexts, the proximal lines were extended orally to the interdental contacts. The finishing and polishing of the preparations were finally performed with the above-mentioned burs and discs.

### 2.2. Manufacturing of the Dental Veneers

**Figure 2:**
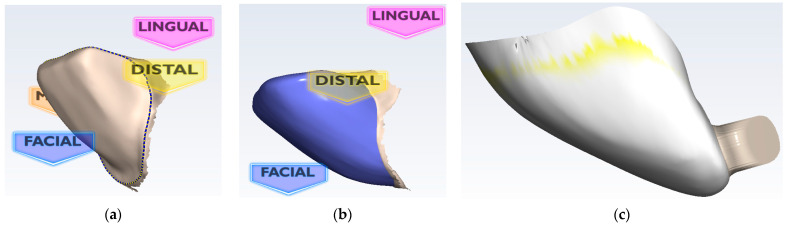
The main steps of designing CR veneers using the Planmeca Romexis Software 4.6.2R: (**a**) tracing the outline of the preparation; (**b**) establishing the design of the veneer; (**c**) final analysis before commanding the milling process.

All sixteen veneers were manufactured using lithium disilicate ceramic (IPS e.max, Ivoclar Vivadent, Liechtenstein) by employing CAD/CAM technology. The chairside Planmeca FIT^®^ system (Planmeca OY, Helsinki, Finland) was used for all of the steps of the restorative workflow, from scanning the preparations to designing, and finally to milling the ceramic veneers. Thus, the scanning procedure, also known as digital impression, was performed with a Planmeca PlanScan Intraoral Scanner (Planmeca OY, Helsinki, Finland). Then, the captured 3D images were built together, and 3D models of the CO and CR preparations were generated. Further on, designing the prospective veneers consisted of outlining the linear or sinusoidal marginal contour and creating the anatomical morphology that simulated the substituted natural teeth (Figure 2).

**Figure 3:**
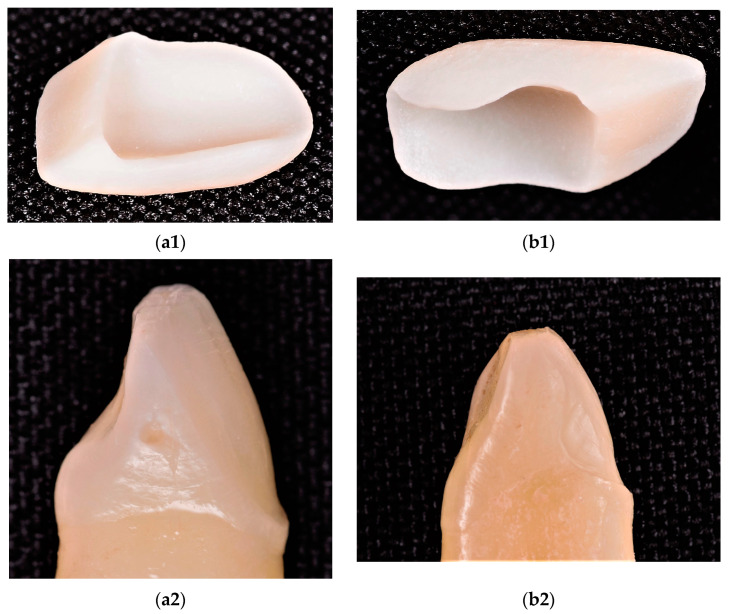
(**a1**,**b1**) The final milled ceramic veneers and (**a2**,**b2**) types of dental preparation: (**a1**,**a2**) CO and (**b1**,**b2**) CR.

The milling sequence was the final step of the technological process; it was performed with the Planmeca PlanMill 40S machine (Planmeca OY, Helsinki, Finland). The selected milling characteristics were: two spindles for enhanced milling speeds, 4 axes, 80 krpm milling speed, and wet processing. The average milling time was 8 to 10 min. The lithium disilicate glass-ceramic blocks utilized for the milling process (IPS e.max CAD HT Monolithic, Ivoclar Vivadent, Schaan, Lichtenstein) streamlined the fabrication of full-contour restorations, with durability, proven clinical properties, as well as excellent esthetics and a high strength of 530 MPa [22]. The final step was the crystallization of the veneers in the PRO-GRAMAT P510 oven (Ivoclar Vivadent, Schaan, Lichtenstein), using the P161 program. The crystallization and glazing procedures were performed according to the manufacturer’s specifications (Figure 3).

All sixteen ceramic veneers were luted to the enamel surface of the preparations. Prior to the luting procedure, the enamel surface and the inner side of the ceramic veneers were conditioned. Thus, the dental structure was etched with 37% orthophosphoric acid (Eco-Etch, Ivoclar Vivadent, Schaan, Lichtenstein) for 20 s, then abundantly washed for 10 s and gently dried without desiccation for another 10 s. The inner side of the veneers was conditioned by applying 9% hydrofluoric acid (IPS ceramic etching gel, Ivoclar Vivadent, Schaan, Lichtenstein) for 20 s, then thoroughly washed for 10 s, and air-dried for another 10 s. In order to promote the bond between the adhesive cement and the ceramic restoration, a primer (Monobond Plus, Ivoclar Vivadent, Schaan, Lichtenstein) was applied on the etched ceramic surface for 1 min and then air dried. Having both the enamel and the ceramic appropriately conditioned for the luting procedure, the bonding agent (Adhese Universal, Ivoclar Vivadent, Schaan, Lichtenstein) was brushed onto both surfaces and then smoothly air-dried. The luting composite (Variolink Esthetic LC, Ivoclar Vivadent, Schaan, Lichtenstein) was carefully applied on the inner side of the veneer, and the restoration was accurately placed on the dental preparation (Figure 3). Cement excess removal was facilitated via a short initial light activation of 3 s, using Bluephase Style curing light (Ivoclar Vivadent, Schaan, Lichtenstein).

The entire light-curing process was then performed for 40 s on the palatal and buccal surfaces of the restored tooth. The polymerization unit had a light intensity of 1000 mW/cm^2^, and its tip was in contact with the surface of the specimens during the entire process of light curing.

### 2.3. The Debonding Tests

Before performing the debonding test, each sample was stored in a 0.9% sodium-chloride physiologically sterile solution at 25 °C so dehydration was avoided.

**Figure 4:**
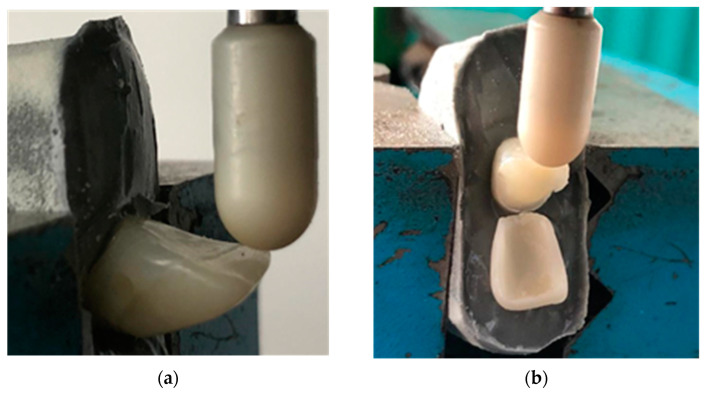
Performing the debonding test: (**a**) the force is applied in the middle of the incisal edge; (**b**) the ceramic veneer is (finally) completely detached.

Considering the specific geometry of the teeth, a dedicated setup was prepared. Thus, each tooth was fixed using a commercial adhesive based on epoxy resin, Epoxyd-Minutenkleber (Weicon, Münster, Germany), in a mold that ensured the parallelism of the fixed surfaces. The adhesive was chosen so that the temperature rise during its exothermic reaction was as low as possible, while its rigidity was not too high. The entire assembly (i.e., tooth and mold) was then fixed using a precision vise (Figure 4). The load was applied on the incisal margin of the palatal surface, perpendicular to the long axis of the tooth and respecting its symmetry axis, using a punch coated in a ceramic material, in order to simulate the real contact conditions.

In the literature, we identified two types of such tests: a shear-flexural test published by Zarone et al. [2] and a test consisting of the application of a compression load on the incisal edge until fracture (load-to-failure test), utilized by Gresnigt et al. [4]. These tests were not standardized, and their settings were not regulated—the first one used a loading speed equal to 0.5 mm/min, and the second one used 1 mm/min. In particular, we considered the failure of the preparation through detachment/debonding of the veneer, without fracture. Thus, we decided to adopt the loading configuration used in [2], which corresponds to our purpose, and a loading speed equal to 1 mm/min. In this configuration, the load is applied on the incisal margin, normal to the palatal surface. Such a force is not one of compression, looking at the assembly of veneer–adhesive–tooth in a global way. Thus, our test is not a regular peel test, considering the complexity of the assembly geometry. It can be named a *failure test by debonding of the veneer*. Referring to the adhesive joints, throughout the study, we use the term *peeling tension* from the considerations presented by Dillard [23].

The tests were performed under the same environmental conditions of temperature, relative humidity, and air pressure in order to avoid the risk of bias error. A Zwick/Roell Z005 testing machine with a 5 kN maximum load was utilized. A loading rate of 1 mm/min and a preload of 5 N were applied. The test was considered completed when the ceramic veneers were completely detached. The values of the force and of the corresponding displacements were recorded throughout all the performed tests.

The geometry of the veneer-tooth assembly varies depending on different parameters (i.e., morphology and dimensions of both tooth and veneer, as well as adhesive surface area) and may impact the recorded values. As a consequence, the normal distribution hypothesis was applied in order to obtain relevant results.

The adhesive surfaces, represented by the vestibular face of the teeth and by the inner surface of the ceramic veneers, were photographed after performing the mechanical tests in order to determine the type of debonding and whether it was cohesive, adhesive, or mixed. Thus, a DSLR camera (Nikon D5500, Tokyo, Japan) and a compact tele macro lens (Tokina ATX M100 f/2.8 PRO D Macro 1:1, Tokyo, Japan) were utilized. Due to its performant features, the macro lens is capable of life-size (1:1) reproduction at a 300 mm distance. As this experimental study was focused on the biomechanical properties determined by two different tooth preparations and not on the imagistic evaluation of the adhesive surfaces, photographing the areas of interest was enough to draw conclusions regarding the type of veneer debonding.

### 2.4. Analytical Evaluation of the Mechanical Strength

**Figure 5:**
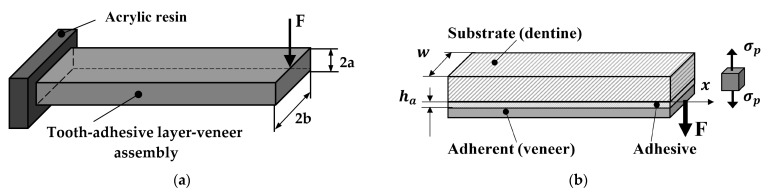
Assumptions made regarding loaded teeth: (**a**) clamped beam loaded in bending with shear force (own sketch adapted from [2]); (**b**) adherent loaded by applied force (own sketch adapted from [23]).

The geometry of the tooth-adhesive layer-veneer assembly is complex. Therefore, it is difficult to analytically assess the stress at failure. Such an attempt implies the acceptance of certain simplifying assumptions. Thus, Zarone et al. considered the assembly as a clamped beam [2], with a rectangular cross-section loaded in bending and shear (Figure 5). The maximum values of the shear and flexural strength were calculated in this study using formulas from the mechanics of materials.

In this paper, the solution proposed by Dillard for the stress distribution of a beam on an elastic foundation was adopted [23]. The relationship in Equation (1) is directly applicable to adhesive bonds. It has a good accuracy when the adhesive is relatively flexible compared to the adherent, as in the case of tooth preparation (Figure 5). For a concentrated force *F* applied at the end of a long-bonded strip (with a length > 5/*β*), the distribution of the peeling stress *σ*_p_ on the symmetry axis *x* of the beam is

(1) $$\sigma_{p}(x)=\frac{E_{a}}{2\cdot h_{a}\cdot E_{v}\cdot I{\cdot \beta }^{3}}{\cdot e}^{-\beta x}\cdot F\cdot \cos\left(\beta x\right)$$

where

(2) $$\beta =\sqrt[{4}]{\frac{E_{a}\cdot w}{4\cdot E_{v}\cdot I{\cdot h}_{a}}}$$

In Equations (1) and (2), *E*_a_ is the Young’s modulus of the adhesive, which is assumed to be linearly elastic, while *E*_v_ and *I* are the Young’s modulus and the centroidal moment of inertia of the veneer, respectively. The *x* coordinate represents the distance along the height of the preparation, measured from the incisal edge.

The thickness *h*_a_ of the adhesive layer and the width *w* of the preparation were considered constant. These two dimensions were determined as means of the values measured at cervical and incisal levels for the adhesive thickness and for the mesial–distal diameter of the tooth, with a 0.01 mm accuracy, as presented in Table 1.

From Equation (1), for *x* = 0, the maximum stress *σ*_p max_ is obtained as:

(3) $$\sigma_{pmax}=\frac{E_{a}}{2\cdot h_{a}\cdot E_{v}\cdot I\cdot \beta^{3}}\cdot F$$

The elastic properties of the materials, Young’s modulus *E*, and Poisson’s ratio *ν* were imported from the literature [23]. They are listed in Table 2. The obtained results, in terms of maximum stress *σ*_p max_, are provided in the next section.

### 2.5. Finite Element Analysis (FEA)

In order to validate the adopted analytical solution, numerical analyses were performed using the finite element method for two of the investigated preparations, previously pointed out in Table 1, randomly selected from each study group: CO3 and CR1. The geometric models for veneers and teeth were obtained by converting into solids the meshes obtained from the 3D scanning. The geometric models were positioned accordingly, leaving 0.5 to 0.6 mm gaps between the veneer and teeth models. These gaps correspond to the maximum values determined in our previous study [21], performed using optical microscopy (for marginal widths of the dental adhesive) and micro-CT (for internal widths of the adhesive). Thus, the most disadvantageous situation was considered for the FEA, as the peak values of the adhesive widths were considered constant throughout the entire surface of the veneer. Furthermore, this width was chosen in order to avoid the penetration between veneer and tooth in the numerical model.

**Figure 6:**
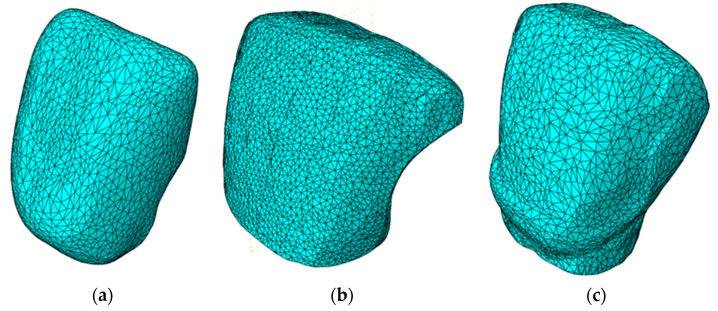
Meshes for: (**a**) veneer; (**b**) adhesive layer; (**c**) central incisive tooth.

The gaps were filled with solid bodies using Boolean operations; the resulting models corresponded to the adhesive layers (Figure 6).

Further on, the models were imported in the commercial software Simulia Abaqus 2019 (Dassault Systèmes SE, Vélizy-Villacoublay, France) and meshed using first-order tetrahedral elements C3D4 (which represents the label of the tetrahedral finite element type utilized in the performed numerical analyses). For the veneers, the average element size was set to 1 mm. The meshes consisted of 28,531 finite elements for the CO3 model and of 25,804 finite elements for the CR1 model. The adhesive layers used an average element size of 0.2 mm. This resulted in 12,160 elements for the CO3 preparation and in 15,715 elements for the CR1 version. The incisors were meshed using an average finite element size of 1 mm. This determined 58,065 elements for the CO3 preparation and 68,591 for the CR1. As an example, the meshes for the CR1 preparation are presented in Figure 6.

In order to replicate the experimental procedure, the simulated load was applied using a spherical indenter with a radius of 1 mm, which was meshed using 656 first-order triangular rigid elements R3D3. The indenter was positioned in the median plane of the models, in contact with the incisal edge of the veneer. The contact between the indenter and the veneer used a surface-to-surface interaction with “hard contact” normal properties and “penalty” tangential properties, using a friction coefficient of 0.2.

**Figure 7:**
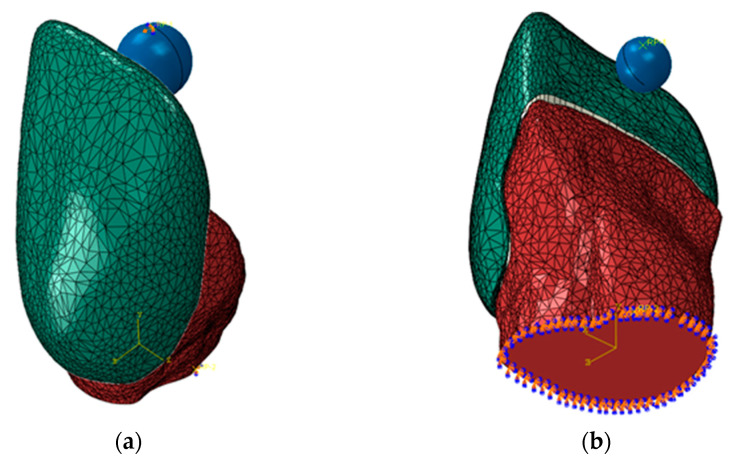
Boundary conditions for (**a**) the CO3 and (**b**) the CR1 preparation.

As far as boundary conditions were concerned, the bottom surface of the incisors was fixed, and the indenter was subjected to a force equal to the value recorded experimentally (Figure 7): 93.90 N for the CO3 preparation and 68.71 N for the CR1 preparation.

**Figure 8:**
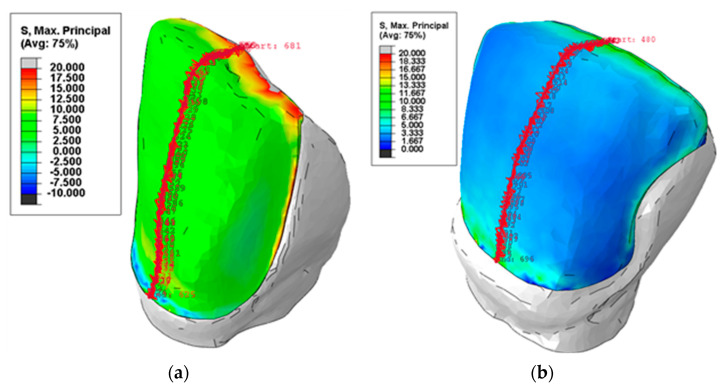
The maximum principal normal stress distributions *σ*_1_ and the node paths for (**a**) the CO3 and (**b**) the CR1 preparation.

The numerical analyses were performed in static conditions, using an implicit solution procedure. As output data, nodal reactions and displacements were recorded for the entire model. The components of the stress were recorded only for the adhesive layer, which was the area of interest. In Figure 8, the distributions of the maximum principal normal stress σ_1_ are plotted, displaying the distribution and intensities of the stress both in color coding and in numerical values.

## 3. Results

The results of the debonding test in terms of values of the maximum force *F*_max_ are presented in Table 3. The Shapiro–Wilk test demonstrated that the highest force values of the two groups have an approximately normal distribution, as long as the *p*-values are higher than 0.05. In this respect, for the CO preparation, the *p*-value was 0.162, while for the CR preparation, it was 0.728. Furthermore, Table 3 presents the mean values, the standard deviations, and the confidence interval for the means of both study groups.

The experimental data demonstrated that the recorded maximum force increased by 14.07% for the CR preparation. However, the maximum force represents an absolute value, which depends on the type and geometry of the teeth. In conclusion, an approach in specific terms, such as the maximum principal normal stress σ_1_ is more appropriate [2].

**Figure 9:**
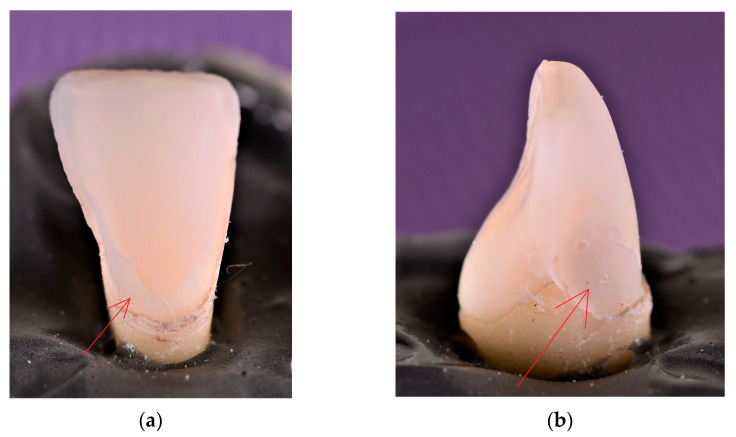
The buccal face of the CO preparation after veneer debonding, displaying the remnant adhesive layer (i.e., the whitish surface highlighted with a red arrow) along its (**a**) cervical and (**b**) distal margins.

The analysis of the macroscopic photographs demonstrated that the debonding was 100% adhesive for both the CO and the CR Groups, as the failure occurred within the interfacial areas, with the adhesive layer remaining either on the tooth surface or on the ceramic veneer. However, there were few differences between the two groups as far as the localization of the remnant adhesive layer was concerned. For the CO samples, the remnant adhesive layer on the tooth surface was mainly observed along its cervical area and its medial and distal third of the buccal face (Figure 9).

**Figure 10:**
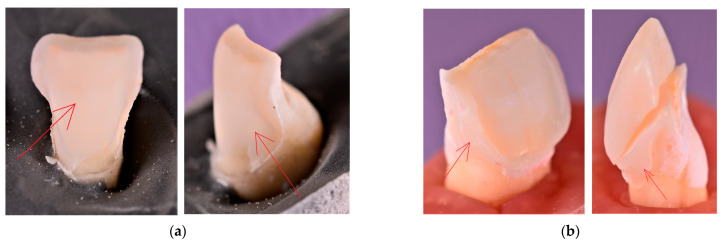
(**a**) The remnant adhesive layer (i.e., the whitish surface highlighted with a red arrow) covering almost entirely the buccal face of the CR preparation; (**b**) the buccal face of one of the CR preparations, displaying the remnant adhesive layer and a fragment of the fractured ceramic veneer (pointed out with a red arrow) along the sinusoidal distal margin.

On the other hand, the tooth surface was almost entirely covered with the adhesive layer after veneer debonding for all CR samples (Figure 10a). In addition to this, a CR veneer fracture was registered, with the ceramic fragment remaining luted to the tooth surface in the immediate proximity of the distal marginal preparation (Figure 10b).

The results of the experimental testing, in terms of maximum stress *σ*_p max_, are listed in Table 4. The Shapiro–Wilk test demonstrated that the maximum stress *σ*_p max_ of the two groups have an approximately normal distribution, as long as the *p*-values are higher than 0.05. In this respect, for the CO preparation, the *p*-value was 0.302, while for the CR preparation, it was 0.867. Furthermore, Table 4 presents the mean values, the standard deviations, and the confidence interval for the mean for both study groups.

The results demonstrate that the maximum stress increased with 25.06% for the CR preparation. By expressing the results in terms of stress, the scattering of data decreases and the values of the standard deviation are approximately equal.

**Figure 11:**
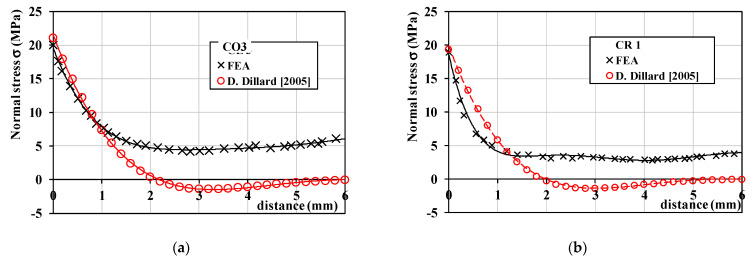
The normal stress distributions for: (**a**) the CO3 preparation; (**b**) the CR1 preparation.

Figure 11 presents the stress distributions obtained for the CO3 and CR1 preparations by using the analytical solution, expressed by Equation (1), as well as by performing the numerical analysis. For the analytical solution, the results are presented in terms of stress *σ*_p_, while for the numerical analysis, the results are presented in terms of maximum principal normal stress *σ*_1_, i.e., the highest value of the normal stress produced in the adhesive preparations.

It can be noted that the normal stress distributions were similar over the distance of 1 mm, starting from the incisal edge. This was precisely the region of the failure initiation. In addition to this, the determined maximum stress values were approximately equal by using the two methods:

- For the CO3 preparation, the relative error was equal to 5.13% (the maximum stress *σ*_p max_ was equal to 21.06 MPa, while the maximum normal principal stress *σ*_1_ reached 19.98 MPa);
- For the CR1 preparation, the relative error was equal to 2.46% (the maximum stress *σ*_p max_ was equal to 19.44 MPa, while the maximum normal principal stress *σ*_1_ reached 18.96 MPa).

Therefore, even if the analytical solution slightly overestimates the maximum stress values, it is validated by the numerical analysis. Moreover, it has the advantage of being easily applicable.

Finally, the maximum debonding stress values presented in Table 4 were analyzed statistically. Since both groups had an approximately normal distribution (verified through the Shapiro–Wilk test, Section 2.4) and equal variances are assumed (the Levene’s test for equality of variances provided a significance equal to 0.893 for an F value of 0.019), a *t*-Test of the difference of the means was performed, according to [24]. In this case, the null hypothesis stated that the means of the maximum normal stress values of the two groups were equal. The summary of the *t*-Test is presented in Table 5, for a confidence level of 0.95.

**Figure 12:**
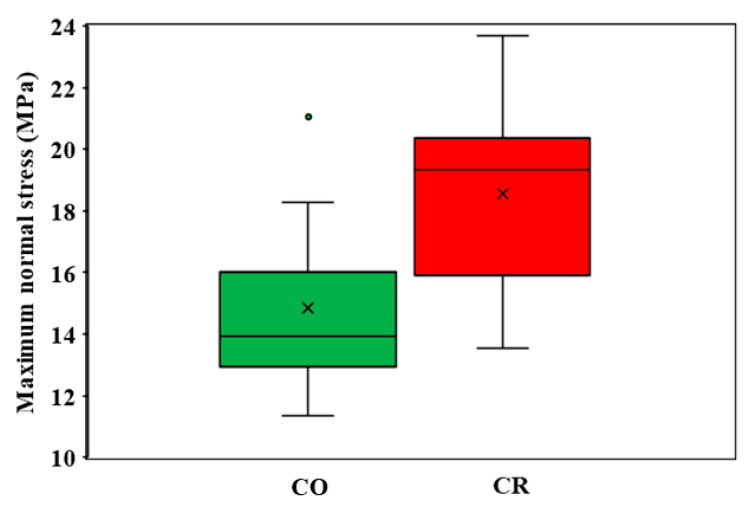
Boxplot of the maximum normal stress reached for the CO and CR samples.

Since the absolute value of −2.236 was higher than the critical **t** of 2.145, the null hypothesis was rejected, and the research hypothesis was accepted; therefore, the means are different. The probability of rejecting was equal to 0.042, when in fact, the null hypothesis was true. Thus, the mean of the maximum normal stress value was 18.57 ± 2.59 (MPa) for the CR tooth preparation and 14.85 ± 2.62 (MPa) for the CO tooth preparation (Figure 12), which demonstrated a significant increase of 25.06% for the CR samples.

## 4. Discussions

Several experimental studies have focused on the correlation between the type of tooth preparation, luting materials, and failure mode of ceramic veneers in order to identify clinical and technical solutions to veneer debonding [25,26,27,28,29,30,31,32,33,34]. However, there are no studies that analyzed the influence that a novel sinusoidal marginal contour, such as the one we have proposed [18], may have on the adhesive interfacial forces. The CO design of the veneers is always characterized by a linear proximal contour that has the advantages of simplicity and reproducibility. However, according to the literature [31,32], this type of design has not improved the debonding strength during mechanical loading and, consequently, the adhesive properties of the luting systems have not been improved over time.

Therefore, the novel CR veneers have been designed as a practical solution aiming to enhance the resistance to the mechanical stress that occurs during functional loading. Their sinusoidal marginal contour provide an interlocking joint between enamel and ceramic, which is deemed to enlarge the contact surface and, subsequently, to determine higher adhesive forces. Moreover, the resulting micro-retentions assure a higher retention of the veneers on the dental substrate and a more accurate positioning of the prosthetic restoration during the luting procedure [21]. In addition to this, the sinusoidal line of the adhesive interface is expected to ensure a higher aesthetic result than the CO linear contour. This is of paramount importance in cosmetic dentistry.

As far as the tooth morphology is concerned, the palatal concavity and the incisal areas of maxillary anterior teeth are considered to be high-stress concentration areas during tooth functioning [33,34]. Whenever the enamel is reduced during the tooth preparation, it should be substituted by a material that has enamel-like properties in order to restore the original biomechanical behavior of a tooth [35]. The long-term success of a prosthetic restoration closely depends on the preparation design and geometry.

High-quality adhesion can be obtained when at least 50% to 70% of the enamel surface is available for the etching procedure [36]. The luting procedure creates two interfaces: the first one is between the ceramic veneer and the composite resin luting agent and the second one is between the composite resin luting agent and the enamel. In order to obtain a long-lasting adhesive joint, an accurate conditioning of both the enamel and the veneer is of paramount importance. Enamel is a dry substrate without vital structures, which contains 92% of the volume of mineral phase (hydroxyapatite); therefore, it is considered the ideal substrate to form a tight adhesive joint. The acid-etch technique is still the gold standard for bonding resin-based materials to the tooth structure. The diffusion and interlocking of resin monomers into the array of micro-porosities left by the acid dissolution of enamel represent the fundamental process of the micromechanical interaction between adhesives and enamel. Bonding to enamel after etching with phosphoric acid is certainly the foundation for the durability of the adhesive restorative procedure [37].

The development of dental adhesive systems has had a significantly positive impact in restorative dentistry. Minimally invasive dentistry focuses on the application of systematic preservation of the original tooth substrates, with the aim to utilize as much enamel as possible. The subsequent restorative procedure often relies on bonding to the remaining tooth structures using an adhesive system with a resin composite [38].

However, the adhesive interfaces have their own issues. Thus, they are prone to microleakage, staining, and secondary decays, as a consequence of inappropriate tooth preparation, veneer fabrication, internal and marginal adaptation of the restoration to dental structure, or of functional and parafunctional forces [24,39,40]. Tribst et al. [41], as well as Andermatt et al. [42] studied the adhesive strength of the composite resin bound to both etched enamel and etched ceramic. The results displayed proper adhesive properties of the tooth/composite and resin cement/enamel interfaces, even if the stress distribution was not uniform because of the combination of plastic and elastic deformation of adhesive, composite resin cement, and enamel [43]. According to other authors, the fracture energy mostly concentrates between the resin cement and the enamel. Consequently, the shearing stress impacts the veneer slide, concentrating compression stress in the weakest areas (i.e., incisal or gingival margins) [44]. This leads to micro-cracks within the adhesive layer, which is responsible for the veneer detachment or fracture. Thus, it must be noted that most fractures are caused by adhesive failure at the porcelain/cement interface [45].

In order to assess the influence that the novel CR design has on the debonding strength, debonding tests were performed in the present work using a Zwick/Roell Z005 universal testing machine. By applying the load on the incisal margin of the palatal surface, perpendicular to the long axis of the tooth, sixteen veneers (divided into two equal groups, CO and CR) have been detached from the enamel surface. The experimental data demonstrated that the recorded maximum force increased by 14.07% for the CR preparation. Therefore, the novel design achieved a significant increase of the adhesive interfacial forces, which is a favorable premise for a long-term prognosis of prosthetic treatments with ceramic veneers.

Imagistic assessments of the debonding surfaces after veneer detachment provided valuable information about the fracture mode. Adhesive failures between the luting cement and ceramic (with the remnant adhesive layer covering the entire tooth surface) were observed for all CR samples. On the other hand, as far as the CO group was concerned, the fracture type was adhesive as well. However, the localization of the remnant adhesive layer was either on the tooth (i.e., mainly along the cervical area and the proximal thirds of the buccal surface) or on the inner side of the ceramic veneer. The adhesive type of interfacial fracture that occurred in both experimental groups highlights the importance of accurate conditioning of the enamel during the luting procedures. This is due to the fact that the quality of the micromechanical interlocking of the resin tags within the micro-porosities in acid-etched enamel has a major impact on the interfacial adhesive forces. Higher bonding forces between the enamel and the adhesive layer were observed in the CR samples, as the remnant adhesive layer covered the entire surface of the tooth. In one of the cases, a fragment of the fractured CR veneer remained bonded along the sinusoidal adhesive joint.

The tooth-adhesive layer-veneer assembly is complex, because it combines different biomechanical properties and surface characteristics of the components. Moreover, there is a large variety of tooth dimensions and shapes that may significantly impact the results of the mechanical tests. In order to perform the analytical evaluation of the mechanical strength, the solution proposed by Dillard for the stress distribution of a beam on an elastic foundation was adopted in the current work [23]. The results of the Shapiro–Wilk test demonstrated that the maximum stress increased by 25.06% for the CR preparation, considering the fact that the maximum debonding stress *σ*_p max_ of the two groups has an approximately normal distribution, as long as the *p*-values are higher than 0.05 (for the CO preparation the *p*-value is 0.302, while for the CR preparation it is 0.867). The results triggered by the debonding test demonstrated higher adhesive forces for the CR samples. Thus, this novel variant proves to be a feasible premise for better clinical performance in oral prosthetic rehabilitation.

The characterization of the mechanical properties and of the stress distribution were important aspects in the FEA, which is a numerical study that shows stress distribution patterns and, consequently, potential fracture areas. Ceramic veneers represent the most appropriate prosthetic solution for aesthetic and functional rehabilitation of anterior teeth. The proper morphology and length of incisal edges facilitate correct anterior guidance and, therefore, the determining factors for enhancing the mechanical behavior of veneers are significant, particularly the preparation design [34,46,47].

The load was applied in the present study by using a spherical indenter on the palatal side of the incisal edge of the veneers, in the middle plane of the teeth. Thus, the experimental procedure during the debonding test was simulated. Its comparative effect on the resulting stresses in CR vs. CO adhesive layers has not been evaluated in previous studies that utilized FEA [34,46,47,48,49]. As is well-known, ceramic has a higher modulus of elasticity than dental tissues and resin composite cement. In contrast, the cement layer behaves similar to a stress absorber. As the aim of this investigation was to determine the influence of the novel preparation design on the stress distribution within the adhesive layer (which was the area of interest), the FEA determined that the normal stress distributions were similar over the distance of 1 mm. The fracture line started from the incisal edge to the cervical margin and along the middle plane, for both the CR and CO exemplified samples. A good match was obtained for this distance between numerical (i.e., FEA) and analytical results.

After analyzing the results, the following conclusions were drawn:

- An accurate adhesive protocol of bonding ceramic veneers to enamel triggers a resistant micromechanical interlocking of the resin tags within the array of micro-porosities in acid-etched enamel. This leads to higher adhesive forces within the enamel–adhesive interface than within the ceramic veneer–adhesive interface.
- In the CR group, the remnant adhesive layer was dispersed over the entire surface of the tooth. This demonstrated that the CR preparation generated higher bonding forces within the enamel–adhesive interface than the CO preparation. Moreover, the fragment of the fractured CR veneer that remained bonded along the sinusoidal adhesive joint displayed valuable information about the influence that the peripheral micro-retentions had over the bonding strength (Figure 10).

A disadvantage of the novel, crenelated design that we have proposed [18] and analyzed in previous studies [20,21], as well as in the present one includes the fact that the dental preparation technique can be quite difficult for unexperienced doctors. Therefore, further research activities must be focused on elaborating a kit of burs specially designed for CR preparations. This is deemed to make the process of dental preparation predictable and repeatable. Furthermore, regarding both CO and CR designs, inappropriate marginal and internal adaptation of the ceramic veneers to enamel may cause microleakage and, thus, a susceptibility to tooth sensitivity, secondary decays, marginal discoloration, and eventually, treatment failure. Hence, investigations such as those carried out on in our previous study are necessary to assess these issues [21]. Contraindications of the proposed crenelated veneers are related to microdontia or severely used teeth due to bruxism or other pathologies; they may cause an insufficient height for properly preparing the sinusoidal proximal margins. Moreover, rotated teeth with one of the proximal margins localized orally to the adjacent tooth trigger difficulties in preparing sinusoidal proximal margins.

Regarding other aspects that could be seen as disadvantages, we must point out that the proximal reduction for CR veneers follows the same steps as for the CO ones; namely, the access to the proximal zone is facilitated by placing a wedge and a metallic matrix between the prepared and the adjacent tooth. Additionally, the CR preparation is as conservative as the CO one because a mock-up is used as a guide for tooth preparation, thus respecting the same preparation steps and techniques available for CO veneers. The only difference between the two types of preparations is the design of the proximal contour. As far as the stress concentrating area is concerned, the FEA analysis in this study demonstrated that the normal stress distributions are similar for CR and CO veneers. Furthermore, the propagation of the adhesive layer fracture was initiated at the incisal edge and continued along the middle line of the facial surface.

Furthermore, the sinusoidal peripheral contour is deemed to provide more accurate in situ positioning of the veneers during the luting procedure, as well as a more appropriate color adaptation along the adhesive interfaces. All these aspects are the subject of future work. Additionally, further in vivo studies are considered to assess the clinical performance of the CAD-CAM of CR veneers, such as adhesive properties and subsequent resistance to debonding and fracture of prosthetic restorations. Finally, all-ceramic systems utilized in dentistry are extremely versatile, various, and comprehensive. The same goes for the technological tools and protocols that are continuously evolving in order to trigger better functional and mechanical properties of the veneers. As a result, further clinical research is planned, with the aim to obtain relevant information for the dentists to apply in their clinical practice.

## 5. Conclusions

Within the limitations of this study (and of the novel proposed design) pointed out above, the following conclusions were drawn:

(i) Crenelated (CR) ceramic veneers, with their proximal sinusoidal contour, provided peripheral micro-retentions that form precise intricate joints between the ceramic veneers and the subjacent enamel. These joints considerably augment the bonding strength and the mechanical properties of the restorations. Thus, CR veneers displayed a higher bonding strength, as the recorded maximum force increased by 14.07%;
(ii) In addition to this, the sinusoidal marginal contour assures a better dental support for the adhesive layer, although the type of fracture was adhesive for both experimental groups. The fact that all CR and CO veneers were detached from the dental preparations due to the interfacial adhesive type of fracture enhanced the importance of accurate conditioning of ceramic and enamel during the luting procedure;
(iii) The maximum peeling stress increased by 25.06% for the CR preparation, which demonstrated the higher adhesive strength of this solution. The remnant adhesive layer covering almost the entire surface of the CR samples after veneer debonding underlines the superiority of the novel, sinusoidal preparation. However, the FEA analysis demonstrated that the preparation design has no influence on the propagation of the adhesive layer fracture;
(iv) Thus, the FEA displayed a similar propagation of the adhesive layer fracture for both study groups. Moreover, the normal stress distributions were similar over the distance of approximately 1 mm starting from the incisal edge, and the determined values of the maximum stress were approximately equal;
(v) An analytical solution for the stress distribution of a beam on elastic foundation was adopted and applied, evaluating the maximum normal stress at failure. Furthermore, it was validated in the case of veneers debonding through the FEA.

## 6. Patents

The novel concept of crenelated veneers is the subject of the Patent No. 131840 B1, with the title *Crenelated dental facets (in Romanian)*, released by OSIM Bucharest, Romania on 30.12.2020.

## Figures and Tables

**Table 1:** Dimensions of the teeth preparations—for each of the considered sample.

Type	*h*_a_ (mm)	*w* (mm)	Type	*h*_a_ (mm)	*w* (mm)
CO1 (LI ^1^)	0.54	5.88	CR1 (LI)	0.615	5.91
CO2 (LI)	0.63	5.89	CR2 (LI)	0.765	6.12
CO3 (LI)	0.69	6.55	CR3 (CI)	0.655	8.48
CO4 (Ca ^2^)	0.875	8.88	CR4 (CI)	0.69	8.45
CO5 (CI ^3^)	0.70	8.11	CR5 (Ca)	0.61	9.44
CO6 (CI)	0.715	8.24	CR6 (LI)	0.58	5.82
CO7 (LI)	0.69	5.96	CR7 (LI)	0.81	5.02
CO8 (Ca)	0.88	9.72	CR8 (LI)	0.735	6.24

^1^ LI, lateral incisor; ^2^ Ca, canine; ^3^ CI, central incisor.

**Table 2:** Elastic properties of the considered materials for the analytical study.

Material	*E* (MPa)	*ν* (unitless)
Veneer	115,000	0.20
Dentine	18,600	0.31
Adhesive	5000	0.35

**Table 3:** The values of the maximum force recorded during the debonding tests for the two study groups.

Type	*F*_max_ (N)	Type	*F*_max_ (N)
CO1 (LI ^1^)	48.94	CR1 (LI)	68.71
CO2 (LI)	57.29	CR2 (LI)	89.71
CO3 (LI)	93.90	CR3 (CI)	120.94
CO4 (Ca ^2^)	86.34	CR4 (CI)	100.89
CO5 (CI ^3^)	82.34	CR5 (Ca)	143.36
CO6 (CI)	88.51	CR6 (LI)	54.52
CO7 (LI)	78.49	CR7 (LI)	51.64
CO8 (Ca)	96.75	CR8 (LI)	91.81
**Mean value**	**79.07**	**Mean value**	**90.20**
**Standard deviation**	**16.08**	**Standard deviation**	**29.81**
**Interval for the mean ^4^**	**79.07 ± 13.44**	**Interval for the mean**	**90.20 ± 24.92**

^1^ LI, lateral incisor; ^2^ Ca, canine; ^3^ CI, central incisor; ^4^ 95% confidence.

**Table 4:** The values of the maximum stress for the two groups.

Type	*σ*_p max_ (MPa)	Type	σ_p max_ (MPa)
CO1 (LI)	11.36	CR1 (LI)	18.80
CO2 (LI)	15.27	CR2 (LI)	19.89
CO3 (LI)	21.06	CR3 (CI)	20.21
CO4 (Ca)	11.49	CR4 (CI)	15.51
CO5 (CI)	13.41	CR5 (Ca)	23.68
CO6 (CI)	14.48	CR6 (LI)	16.06
CO7 (LI)	18.29	CR7 (LI)	13.54
CO8 (Ca)	13.41	CR8 (LI)	20.84
**Mean value**	**14.85**	**Mean value**	**18.57**
**Standard deviation**	**3.13**	**Standard deviation**	**3.10**
**Interval for the mean ^1^**	**14.85 ± 2.62**	**Interval for the mean**	**18.57 ± 2.59**

^1^ 95% confidence.

**Table 5:** *t*-Test summary for the two types of veneers: CO (Group 1) and CR (Group 2).

Type	*Mean*	*Variance*	*n*	*Pooled Variance*	*df* ^1^	*t* ^2^	*p (T ≤ t)*	*t_critic_*
CO	14.85	11.19	8	11.074	14	−2.236	0.042	2.145
CR	18.57	10.96	8					

^1^ degrees of freedom for the two groups; ^2^ *t*-Test statistic value.

## Data Availability

Data supporting the reported results can be obtained from the first author.
